# Hierarchical Porous Interlocked Polymeric Microcapsules: Sulfonic Acid Functionalization as Acid Catalysts

**DOI:** 10.1038/srep44178

**Published:** 2017-03-16

**Authors:** Xiaomei Wang, Jinyan Gu, Lei Tian, Xu Zhang

**Affiliations:** 1 Department of Polymer Science and Engineering, Hebei University of Technology, Tianjin 300130, P.R. China

## Abstract

Owing to their unique structural and surface properties, mesoporous microspheres are widely applied in the catalytic field. Generally, increasing the surface area of the specific active phase of the catalyst is a good method, which can achieve a higher catalytic activity through the fabrication of the corresponding catalytic microspheres with the smaller size and hollow structure. However, one of the major challenges in the use of hollow microspheres (microcapsules) as catalysts is their chemical and structural stability. Herein, the grape-like hypercrosslinked polystyrene hierarchical porous interlocked microcapsule (HPIM-HCL-PS) is fabricated by SiO_2_ colloidal crystals templates, whose structure is the combination of open mouthed structure, mesoporous nanostructure and interlocked architecture. Numerous microcapsules assembling together and forming the roughly grape-like microcapsule aggregates can enhance the structural stability and recyclability of these microcapsules. After undergoing the sulfonation, the sulfonated HPIM-HCL-PS is served as recyclable acid catalyst for condensation reaction between benzaldehyde and ethylene glycol (TOF = 793 h^−1^), moreover, exhibits superior activity, selectivity and recyclability.

As one important functional polymeric material, polymeric microcapsules have attracted considerable attention due to their unique physicochemical properties^1^,^2^. Their unique properties make them valuable for many potential applications, such as drug delivery^3^, catalytic carrier^4^, controlled release^5^, adsorption and separation^6^,^7^ and stimuli-responsive material^8^. Motivated by their promising prospects, great efforts have been devoted to the fabrication of the polymeric microcapsules, such as template-assisted methods^9^,^10^, self-assembly of block copolymer process^11^,^12^, interfacial mini-emulsion polymerization methods^13^ and microfluidic approach^14^. Among such methods, the template-assisted methods are efficient to prepare polymeric microcapsules with well-defined size and morphology as a result of the desirable stability and monodispersity of the templates^1^,^2^.

For effectively decreasing mass transfer resistance and greatly enhancing catalytic activity of polymeric microcapsules, the following critical structural/morphological characteristics should be taken into consideration: the size and shape of the entire microcapsule; the porosity on the capsule wall; the thickness, structure/morphology, and the stability of the capsule wall^15^,^16^. Compared with the former two issues which are primarily related to mass transfer rate for the substances, the latter one is related to the mechanical stability and recyclability of the microcapsules. Nevertheless, in most cases, the enclosed microcapsules with the dense and thick capsule wall would cause the high mass transfer resistance between the capsule lumen and the bulk solution, thus decreasing the apparent catalytic activity^17^,^18^. As reported previously, the hierarchical porous nanostructure that contain open mouthed (macroporous) structure and mesoporous nanostructure can effectively decrease mass transfer resistance from the capsule wall, improve the flow rate of the solution in the capsule lumen, make fully use the exposed outer and inner surfaces, thus greatly enhance catalytic activity^17^,^18^.

Compared with other kinds of porous microcapsules, porous polymeric microcapsules gradually become an important and attractive class of porous materials owing to the unique properties of large surface areas and high chemical stabilities^19^. As we all know, crosslinking of swollen chloromethylated polystyrene via Friedel-Crafts alkylation is a facile and popular method for formation of the porous structure^19^,^20^. Meanwhile, this hypercrosslinked method can effectively improve mechanical strength and solvent resistance of porous polymeric microcapsules^19^. For enhancing catalytic efficiency, porous polymeric microcapsules with the ultrathin shell are desirable^17^. However, the recyclability of these microcapsules represented a challenge in the field of catalyst. Introducing magnetic property is a proven and effective way to improve the cycling performance of polymeric microcapsules. Unfortunately, the loss of the magnetic properties and poor recyclability still appear during the repeated catalytic runs, which restricts the practical applications^21^. In order to solve this formidable challenge, the interlocked polymeric microcapsules, which integrate numerous microcapsules into a targeted nanocomposite, have attracted intensive attention owing to their excellent recyclability and mechanical stability^22^,^23^. As catalyst supports, we can introduce open mouthed structure and interlocked architecture in the multifunctional polymeric microcapsules. This way may open a novel interdisciplinary area between catalysis and polymer science.

In this paper, we present a facile method to fabricate grape-like hypercrosslinked polystyrene hierarchical porous interlocked microcapsule (HPIM-HCL-PS) by silica colloidal crystals templates (CCTs). Their structure is the integration of open mouthed structure, hierarchical porous nanostructure and interlocked architecture. The presence of interlocked architecture makes numerous microcapsules assemble together and form the roughly grape-like microcapsule aggregates. This interlocked structure endows HPIM-HCL-PS with excellent mechanical stability and recyclability. Hierarchical porous nanostructure contains open mouthed (macroporous) structure and mesoporous nanostructure provided not only large specific surface area for high catalytic activity but also highly developed hierarchical macro/mesoporosity for rapid mass transport. Therefore, hierarchical porous polymeric microcapsules have enhanced properties compared with single-sized porous structure. Herein, as recyclable acid catalysts, the sulfonated HPIM-HCL-PS (HPIM-HCL-SPS) for condensation reaction between ethylene glycol and benzaldehydeas is illustrated as an example. The detailed fabrication process of HPIM-HCL-PS has been illustrated in Fig. 1. Linear polystyrene shells are first grown onto the surface of the silica CCTs by surface-initiated atom transfer radical polymerization (SI-ATRP), functionalized with chloromethyl groups and then hypercrosslinked via Friedel-Crafts alkylation. After the silica CCTs are removed, HPIM-HCL-PS is achieved and followed by a sequential sulfonation resulting in sulfonic groups (HPIM-HCL-SPS). As we all know, liquid acids (e.g., H_2_SO_4_, HF, and H_3_PO_4_) used as catalyst in the acid-catalyzed reactions will cause difficulties in product separation, equipment corrosion and environmental pollution, thus, environmentally recoverable solid acid catalysts are highly desirable. The HPIM-HCL-SPS is served as recyclable acid catalyst for condensation reaction between ethylene glycol and benzaldehyde, moreover, exhibits high catalytic activity.

## Results and Discussion

**Figure 2:**
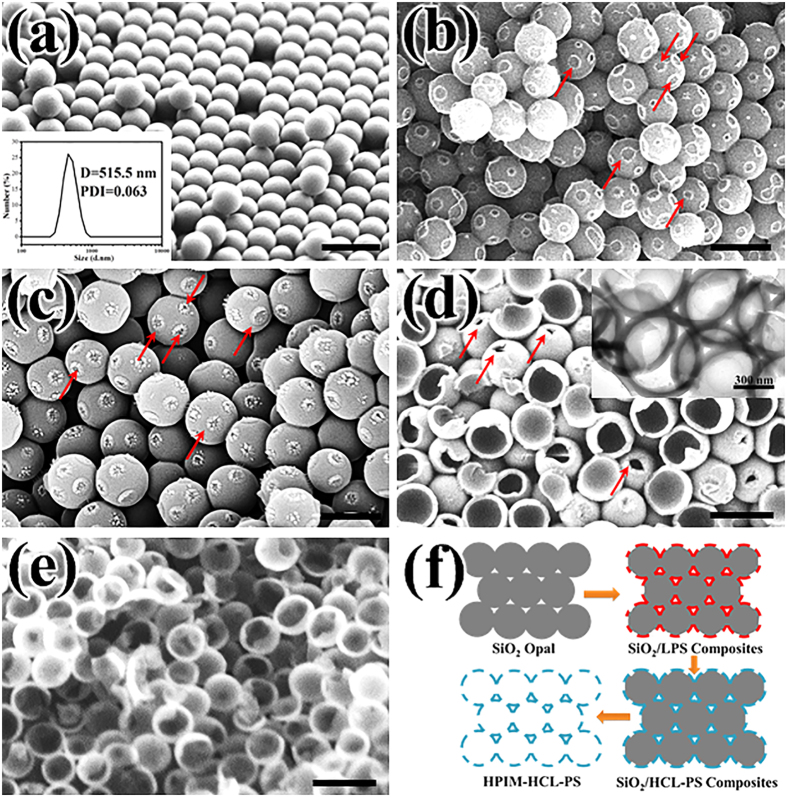
(**a**) SEM image of SiO_2_ opals and average size distri-bution of SiO_2_ microspheres (inset); (**b**) SEM image of SiO_2_@LPS composites; (**c**) SEM image of SiO_2_@HCL-PS composites; (d) SEM (1.00 kV) image and TEM image (inset) of HPIM-HCL-PS; (**e**) STEM (8.00 kV) image of HPIM-HCL-PS; (**f**) schematic illustration of preparation process of the HPIM-HCL-PS. All the scale bars are 1 μm.

The SiO_2_ microsphere with an approximate diameter size of 550 nm that constructed SiO_2_ opals (the sacrificial template) was firstly prepared through Stöber method^24^. The polystyrene ATRP from the silica particles exhibited the characteristics of a controlled/“living” polymerization, had a narrow molecular weight distribution (M_w_/M_n_ < 1.36), the number average molecular weight is 6.33 × 10^4^. The distribution plot was shown in Figure S1 (Supporting Information). The SEM photograph of SiO_2_ opals was shown in the Fig. 2a. It is observed that SiO_2_ opals had a hexagonal close-packed structure, which indicated that the template microspheres had a very uniform shape and exhibited the monodisperse property (Fig. 2a). Subsequently, the Br-containing SI-ATRP initiation sites were introduced on the surface of the SiO_2_ microspheres by BITS^22^. The energy-disperse X-ray (EDX) pattern was shown in the Figure S2 (Supporting Information). The weak but evenly distributed signals from C and N element demonstrated that the silica opal was modified by the BITS. This result further confirmed the existence of ATRP initiation sites on the surface of SiO_2_ microspheres. The SI-ATRP grafting of linear polystyrene (LPS) from the SiO_2_-Br microspheres was carried out to form SiO_2_@LPS composite opals. In the cross-sectional SEM image of the SiO_2_@LPS composite opals (Fig. 2b), we can clearly observe the core/shell structure. The content of PS grafted layer after chloromethylation is about 32% according to the TGA curve (Figure S3, Supporting Information). Moreover, in the Fig. 2b, it is also definitely found that there exist several clear pits on the LPS shell, marked by the red arrow. At the contacting sites, few BITS modified the silica microspheres led to few LPS forming. After hypercrosslinking reaction, the LPS shell became coarse and showed some shrinkage owing to the crosslinking of chloromethylated LPS molecular chains (Fig. 2c). The SEM and STEM images of HPIM-HCL-PS were shown in the Fig. 2d and e, respectively. The ultrathin shell of microcapsules was around 60 nm. These microcapsules were interconnected with each other via the open mouths and presented a grape-like interlocked architecture. During the Friedel-Crafts reaction, the SiO_2_@LPS composite opal structure (numerous microspheres contacted with each other) facilitates the interparticle cross-linking, which makes the final microcapsules interlock with each other by covalent bond and form the interlocked architecture (Fig. 2f)^9^. In principle, each microcapsule should contain twelve open mouths, since every template microsphere is in interconnected with twelve neighboring microspheres in hexagonal close-packing (Fig. 2f)^25^,^26^. Furthermore, the pre-existing open mouths can facilitate the template removal compared to the traditional microcapsules prepared by hard templating method.

For the hypercrosslinked porous polymer, Friedel-Crafts reaction is an excellent method to form mesopores without adding pore forming agents^20^. The permanent porosity in hypercrosslinked porous polymer is a result of extensive crosslinking reactions. This way can effectively prevent the polymer chains from collapsing into a dense, nonporous state^19^. The mesostructure of the HPIM-HCL-PS formed via hypercrosslinking the chloromethylated LPS was confirmed by nitrogen adsorption measurements (Figure S4, Supporting Information). The adsorption and desorption isotherms of the obtained HPIM-HCL-PS show a type II isotherm, indicating the presence of mesopores. However, the adsorption and desorption branches of HPIM-HCL-PS did not close completely in the region of low relative pressure, presumably due to a typical nature of these mesopolymers^27^,^28^,^29^.

**Figure 3:**
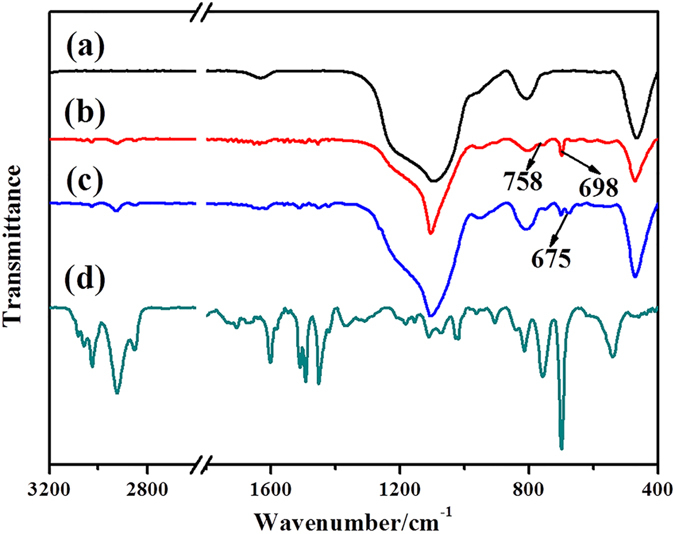
FT-IR spectra of (**a**) SiO_2_ opals, (**b**) SiO_2_@LPS composite opals, (**c**) chloromethylated SiO_2_@LPS composite opals, and (**d**) HPIM-HCL-PS.

The chemical compositions of the corresponding products were also characterized by FT-IR (Fig. 3). In Fig. 3a–c, it can be found that the 1100 cm^−1^ corresponding to a Si-O-Si stretch. After grafting of linear polystyrene from the SiO_2_ microspheres, peaks arising from the C-H vibrating signals of a mono-substituted benzene at 698 and 758 cm^−1^ were observed (Fig. 3b). In the spectrum of the chloromethylated SiO_2_@LPS composites (Fig. 3c), the characteristic bands at 675 cm^−1^ was assigned to the derived chloromethyl groups (-CH_2_Cl), indicating that the chloromethylation reaction successfully occurred in the LPS shells^25^. The FT-IR spectra of HPIM-HCL-PS indicated that almost all the silica templates were removed, as indicated by the disappearance of the characteristic peak at 1100 cm^−1^ (Fig. 3d).

**Figure 4:**
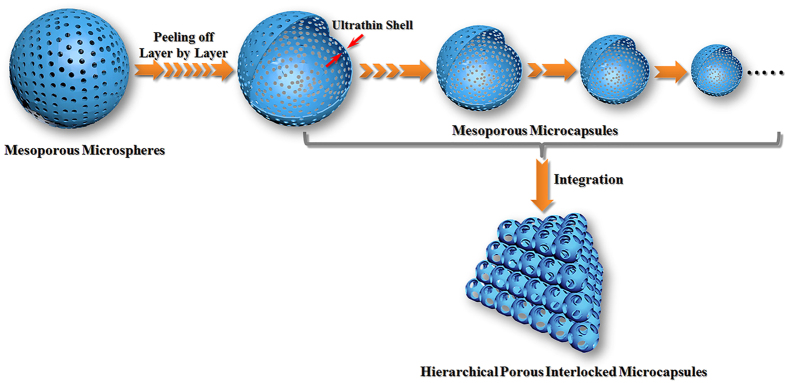
Schematic illustration of HIPM structure.

Mesoporous microspheres have attracted much attention in the field of catalysis due to their unique structural and surface properties. In catalytic reactions, two key factors for the catalytic activity are mass transfer rate as well as utilization ratio of active sites. Compared to the traditional solid mesoporous microspheres, theoretically, the mesoporous microcapsules with smaller size can effectively shorten the transmission path, improve the mass transfer rate and increase the utilization ratio of active sites. Therefore, we propose a model that the solid mesoporous microspheres can be peeled off layer by layer to form numerous smaller microcapsules with ultrathin shell. These microcapsules can provide shorter transmission path, and effectively raise the utilization ratio of active sites (Fig. 4). Meanwhile, preparation of the open-mouthed microcapsules with ultrathin capsule wall is a good choice to further decrease the mass transfer resistance between the capsule lumen and the bulk solution, improve the flow rate of the solution in the capsule lumen. However, these microcapsules are difficult to recycle and reuse, resulting in low catalytic performance due to their weakly chemical and structural stability. Herein, numerous mesoporous microcapsules with ultrathin shell and open mouths were integrated into a targeted nanocomposite (HPIM). In this case, on the premise of guaranteeing the recyclability and mechanical stability, the catalytic activities of these microcapsules were still improved considerably.

To further investigate the relationship between catalytic properties and structural characteristics, the activity of the HPIM-HCL-SPS as the recyclable acid catalysts is evaluated using the condensation reaction of benzaldehyde and ethylene glycol (Table 1). In the HPIM-HCL-SPS spectrum (Figure S5, Supporting Information), the characteristic bands at 1126, 1178 and 1220 cm^−1^ are assigned to the derived sulfonic acid groups (−SO_3_H)^30^. The number of immobilized protons on the HPIM-HCL-SPS is 1.45 mmol/g based on acid-base titration. This H^+^ content is lower than other samples in the literature^21^,^27^, which likely owe to the hypercrosslinking reaction that leads to the sulfonation reaction sites reducing sharply. As shown in Table 1, the conversion of benzaldehyde is around 70% with a high selectivity (98%) toward 2-phenyl-1,3-dioxolane. This conversion is higher than the other catalyst for this condensation reaction^21^. Moreover, on the basis of the number of immobilized protons on the HPIM-HCL-SPS, the average turnover frequency (TOF) was about 793 h^−1^ when the conversion reached 70%. This result is also higher than the literature values for the same model catalysis (Table 2)^21^,^27^. For a fair comparison, the TOFs were calculated based on the H^+^ contents in the system (the same is done for literature values). The outstanding catalytic performance of HPIM-HCL-SPS was attributed to their ultrathin shell and hierarchical porous structure. The open-mouthed structure can effectively improve the mass transfer rate of the capsule lumen and made full use of the exposed outer and inner surfaces. The ultrathin shell greatly shortened the transmission path and increased the utilization ratio of active sites for catalysis reactions. Therefore, the HPIM-HCL-SPS exhibited higher activity in the same reaction time (1 h) though the H^+^ content (1.45 mmol/g) is lower than other samples in the literatures.

Another key parameter to evaluate the catalytic performance is recyclability and mechanical stability of the HPIM-HCL-SPS. After five successive cycles, the HPIM-HCL-SPS was still stable and active, with a conversion of 63.5%. After reaction, the HPIM-HCL-SPS was collected and characterized again with SEM to check their structural stability. As shown in the SEM image (Figure S6, Supporting Information), after five cycles of catalytic tests, the HPIM-HCL-SPS maintained the roughly grape-like morphology and the mesoporous structure hardly changed (Figure S7, Supporting Information). Furthermore, after the fifth cycle, acid-base titration demonstrateed the protons content of HPIM-HCL-SPS (1.2 mmol/g) is still sufficiently high concentration to catalyze the condensation reaction. The above results can be attributed to the existence of interlocked architecture. Interlocked architecture makes numerous microcapsules assemble together, forms the roughly grape-like microcapsule aggregates and thus greatly improves the recyclability and mechanical stability. Moreover, compared to the traditional organic polymeric microcapsules without crosslinking, the highly crosslinked nature of the HPIM-HCL-PS confers them higher chemical and thermal stability.

In summary, the grape-like HPIM-HCL-PS was fabricated by colloidal crystals templates method, whose structure is the integration of open mouthed structure, mesoporous nanostructure and interlocked architecture. Attributed to the structural effect, HPIM-HCL-SPS has exhibited significantly enhanced catalytic activity and recyclability in the condensation reaction of benzaldehyde and ethylene glycol with superior activity and selectivity. Benefiting from the merits of easy functionalization due to the existence of abundant benzene rings, high utilization ratio of active sites and rapid mass transport, excellent chemical stability and recyclability, the HPIM-HCL-PS has tremendous application potential in the catalytic field.

## Methods

### Materials

Chloromethyl ether (CME, Henan Wanxiang Chemical-technical Factory, chlorine content 41%) was dried by calcium chloride. Monodisperse silica microsphere that constructed colloidal crystal template (SiO_2_-CCT) was synthesized according to Stöber method^24^. [3-(2-Bromoisobutyryl) propyl]-trimethoxysilane (BITS) was synthesized according to Yang *et al*.^23^. Styrene (St), dimethyl Formamide (DMF) were distilled under reduced pressure, then stored under argon at −10 °C. N, N, N′, N″, N″-pentamethyldiethylenetriamine (PMDETA, 98%), benzaldehyde and 1,2-dichloroethane (DCE) were purchased from Aladdin Industrial Corporation. All other reagents were used as received.

### Preparation of Silica@Linear Polystyrene (SiO_2_@LPS) Composite Opals

Monodisperse dispersions of silica microspheres were centrifuged at 1000 rpm for 13 h to form the silica opal, then allowed to air-dry^25^. After sintering at 500 °C for 3 h to enhance the interconnectivity between the microspheres, the dried silica opals were heated at 60 °C in the mixture of ethanol, ammonia and deionized water. Then, an excess of BITS was added, and the mixture was kept to reflux for 24 h. Finally, the modified silica opals were washed with ethanol to remove the residual BITS and dried under vacuum^23^.

A glass tube with a magnetic bar was loaded with 0.05 g BITS modified silica opals and vacuum pumped for 2 h. St (1 mL), PMDETA (0.04 mL), DMF (2 mL) and CuCl (17.8 mg) were mixed under an argon atmosphere and stirred until a homogeneous solution was formed. The solution was then quickly injected into the tube containing BITS modified silica opals under vacuum. After the glass tube was filled with argon, the SI-ATRP was carried out at 100 °C for 12 h under slow stirring. The product was washed by DMF, extracted with ethanol for 12 h and dried under vacuum at 60 °C.

### Preparation of HPIM-HCL-PS

SiO_2_@LPS composite opal, CME solution and ZnCl_2_ were introduced into a 50 mL two-necked flask under the argon atmosphere. After the SiO_2_@LPS composite opal was immersed in the mixture at ambient temperature for 2 h, the reaction was carried out at 35 °C for 1 h. The product was washed and extracted with ethanol for 12 h and dried at 60 °C under vacuum. The hypercrosslinking reaction was carried out using 0.1 g dried chloromethylated SiO_2_@LPS composite opal pre-swollen in 10 mL of 1,2-dichloroethane for 12 h. After lewis acid catalyst FeCl_3_ (0.5 g) was added into the reaction mixture under an argon atmosphere, the reaction was carried out at 80 °C for 5 h. The resulting product was filtrated and washed with ethanolic solution of 1 vol% HCl for three times, then extracted with ethanol and subsequently freeze-dried with 1,4-dioxane. Finally, the SiO_2_@HCL-PS composite opals were immersed into aqueous HF (40 wt.%) to etch the silica template. After being washed with water to neutrality, the samples were freeze-dried with 1,4-dioxane.

### Sulfonation of HPIM-HCL-PS and Catalytic Performance Test

0.05 g of the freeze-dried HPIM-HCL-PS was swollen in DCE (3 mL) at ambient temperature for 12 h. After being stirred in an ice bath for 2 h, the mixture consisting of sulfuric acid (98 wt.%, 2.5 mL) dropwise to a solution of acetic anhydride (4.7 mL) in DCE (4 mL) added to the flask loaded with pre-swollen HPIM-HCL-PS. The sulfonation was carried out at 40 °C for 48 h under slow stirring. HPIM-CLPS-SPS were ready for catalytic test after rinsing with water and freeze-dried with 1,4-dioxane.

The condensation reaction of ethylene glycol and benzaldehyde was performed by string the mixture of HPIM-HCL-SPS (0.01 g), benzaldehyde (0.015 mol), ethylene glycol (0.015 mol) and cyclohexane (5 g) for 1 h at 90 °C. To study the catalyst recyclability, the catalyst was washed three times with ethanol to remove the entire residue, freeze-dried with 1,4-dioxane, and then reused for subsequent recycle under the same catalytic reaction conditions. Then the obtained liquid solution was analyzed by SHIMADZU GC-2010 Plus gas chromatograph (GC) equipped with a flame ionization detector working at 280 °C and a 30 m RTX-1 column. The injected samples were heated from 160 to 200 °C at a heating rate of 15 °C/min.

### Characterization

Hydrodynamic diameter measurements were performed on a Malvern Zetasizer Nano-ZS90. Structure and morphology of the samples were characterized using scanning electron microscopy (SEM, FEI Nova Nano-SEM450 at 1–8 kV) with a concentric backscattered retractable (CBS) detector and transmission electron microscopy (TEM, JEM-1011 at 100 kV). The samples for fourier transform infrared spectroscopy (FT-IR) characterization were measured by Bruker VECTOR-22 using potassium bromide (KBr) pellets. The molecular weights and molecular weight distributions of the grafted linear PS were determined using gel permeation chromatography (GPC, PL-GPC-220) in THF at 40 °C with a flow rate of 1 mL/min. The linear PS grafted from SiO_2_ was gained by etching the silica cores, and then purified through precipitation in methanol. Thermogravimetric analysis (TGA) was performed on a SDT-Q600 instrument, at a scan rate of 10 °C/min, up to 800 °C under air. Nitrogen adsorption-desorption isotherms were recorded at 77 K using the ASAP 2020 M + C surface area and porosity analyzer. The Brunauer-Emmett-Teller (BET) method was utilized to calculate the specific surface areas. The pore volume and pore size were derived by using the Barrett-Joyner-Halenda (BJH) model. The amount of acid sites was quantified by an acid-base titration method^21^.

## Additional Information

**How to cite this article:** Wang, X. *et al*. Hierarchical Porous Interlocked Polymeric Microcapsules: Sulfonic Acid Functionalization as Acid Catalysts. *Sci. Rep.* **7**, 44178; doi: 10.1038/srep44178 (2017).

**Publisher's note:** Springer Nature remains neutral with regard to jurisdictional claims in published maps and institutional affiliations.

## Supplementary Material

Supplementary Information

## Figures and Tables

**Figure 1:**
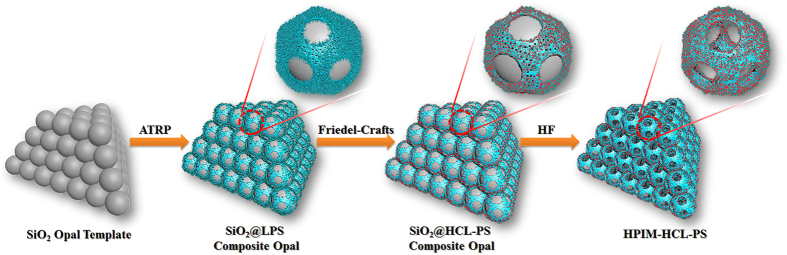
Schematic illustration of preparation process for HPIM-HCL-PS.

**Table 1:** Conversion of benzaldehyde catalyzed by HPIM-HCL-SPS.

Catalyst	Cycle	Benzaldehyde Conversion, %	2-phenyl-1,3,dioxane selectivity, %	Side products, %
Blank	—	35.6	96.2	1.3
HPIM-HCL-SPS	1	62.9	99.2	0.5
	2	69.1	98.8	0.8
	3	60.8	98.5	0.9
	4	76.7	99.1	0.6
	5	63.5	99.0	0.6

**Table 2:** Comparison of the turnover frequency (TOF) in the condensation reaction of benzaldehyde and ethylene glycol as reported in the literature.

Catalyst	TOF (h^−1^)	S_BET_^a^ [m^2^/g]	V_p_^b^ [cm^3^/g]	D_p_^c^ [nm]
HPIM-HCL-SPS	793	604	0.27	3.0
FUD-14-SO_3_H^27^	548	539	0.34	3.2
Fe_3_O_4_@DVB-2-H^21^	433	45	—	—

## References

[b1] Haladjova E. . Polymeric nanoparticle engineering: from temperature-responsive polymer mesoglobules to gene delivery systems. Biomacromolecules. 15, 4377–4395 (2014). 25320910 10.1021/bm501194g

[b2] Lou X. W. (David), Archer L. A. & Yang Z. Hollow micro-/nanostructures: synthesis and applications. Adv. Mater. 20, 3987–4019 (2008).

[b3] Villiers M. M., Otto D. P., Strydom S. J. & Lvov Y. M. Introduction to nanocoatings produced by layer-by-layer (lbl) self-assembly. Adv. Drug Delivery Rev. 63, 701–715 (2011). 10.1016/j.addr.2011.05.011 21699936

[b4] Choi S., Gopalan A. I., Ryu J. & Lee K. P. Hollow spherical nanocapsules of poly(pyrrole) as a promising support for pt/ru nanoparticles based catalyst. Mater. Chem. Phys. 120, 18–22 (2010).

[b5] Abbaspourrad A., Carroll N. J., Kim S. H. & Weitz D. A. Polymer microcapsules with programmable active release. J. Am. Chem. Soc. 135, 7744–7750 (2013). 23607271 10.1021/ja401960f

[b6] Zhang S., Zhou Y., Nie W., Song L. & Zhang T. Preparation of uniform magnetic chitosan microcapsules and their application in adsorbing copper ion (ii) and chromium ion(iii). Ind. Eng. Chem. Res. 51, 14099–14106 (2012).

[b7] Lv Y., Lin Z. & Svec F. Hypercrosslinked large surface area porous polymer monoliths for hydrophilic interaction liquid chromatography of small molecules featuring zwitterionic functionalities attached to gold nanoparticles held in layered structure. Anal. Chem. 84, 8457–8460 (2012). 22998108 10.1021/ac302438m PMC3482311

[b8] Ali S. I., Heuts J. P. A. & Herk A. M. Vesicle-templated pH-responsive polymeric nanocapsules. Soft Matter. 7, 5382–5390 (2011).

[b9] Haladjova E., Rangelov S., Tsvetanov C. & Simon P. Preparation of polymeric nanocapsules via nano-sized poly(methoxydiethyleneglycol methacrylate) colloidal templates. Polymer. 55, 1621–1627 (2014).

[b10] Wu D. . Nanoporous polystyrene and carbon materials with core shell nanosphere-interconnected network structure. Macromolecules. 44, 5846–5849 (2011).

[b11] Choi S. H., Lee S. H. & Park T. G. Temperature-sensitive pluronic/poly(ethylenimine) nanocapsules for thermally triggered disruption of intracellular endosomal compartment. Biomacromolecules 7, 1864–1870 (2006). 16768408 10.1021/bm060182a

[b12] LoPresti C., Lomas H., Massignani M. & Battaglia T. S. G. Polymersomes: nature inspired nanometer sized compartments. J. Mater. Chem. 19, 3576–3590 (2009).

[b13] Cao Z. & Shan G. Synthesis of polymeric nanocapsules with a crosslinked shell through interfacial miniemulsion polymerization. J. Polym. Sci., Part A: Polym. Chem 47, 1522–1534 (2009).

[b14] Abbaspourrad A., Datta S. S. & Weitz D. A. Controlling release from ph-responsive microcapsules. Langmuir. 29, 12697–12702 (2013). 24041287 10.1021/la403064f

[b15] Shi J. . Exploring the segregating and mineralization-inducing capacities of cationic hydrophilic polymers for preparation of robust, multifunctional mesoporous hybrid microcapsules. ACS Appl. Mater. Interfaces 5, 5174–5185 (2013). 23675684 10.1021/am401017y

[b16] Shi J., Zhang X., Zhang S., Wang X. & Jiang Z. Incorporating mobile nanospheres in the lumen of hybrid microcapsules for enhanced enzymatic activity. ACS Appl. Mater. Interfaces. 5, 10433–10436 (2013). 24164487 10.1021/am404210m

[b17] Shi J., Zhang S., Wang X. & Jiang Z. Open-mouthed hybrid microcapsules with elevated enzyme loading and enhanced catalytic activity. Chem. Commun. 50, 12500–12503 (2014). 10.1039/c4cc05809g 25189769

[b18] Mandal S. . Open-mouthed metallic microcapsules: exploring performance improvements at agglomeration-free interiors. J. Am. Chem. Soc. 132, 14415–14417 (2010). 20879769 10.1021/ja107589m

[b19] Xu S., Luo Y. & Tan B. Recent development of hypercrosslinked microporous organic polymers. Macromol. Rapid Commun. 34, 471–484 (2013). 23362134 10.1002/marc.201200788

[b20] Li B., Gong R., Luo Y. & Tan B. Tailoring the pore size of hypercrosslinked polymers. Soft Matter. 7, 10910–10916 (2011).

[b21] Feyen M., Weidenthaler C., Schuth F. & Lu A. Synthesis of structurally stable colloidal composites as magnetically recyclable acid catalysts. Chem. Mater 22, 2955–2961 (2010).

[b22] Liu B., Jin Z., Qu X. & Yang Z. A. General approach toward opal-inverse opal interlocked materials. Macromol. Rapid Commun. 28, 322–328 (2007).

[b23] Rong J., Ma J. & Yang Z. Opal gels templated synthesis of structured titania materials. Macromol. Rapid Commun. 25, 1786–1791 (2004).

[b24] Stöber W. & Fink A. Controlled growth of monodisperse silica spheres in the micron size range. J Colloid Inter Sci. 26, 62–69 (1968).

[b25] Zhang X. . Enhanced adsorption capacity and selectivity towards salicylic acid in water by a cationic polymer functionalized 3-d ordered macroporous adsorbent. Soft Matter. 9, 6159–6166 (2013).

[b26] He H. . Three-dimensionally ordered macroporous polymeric materials by colloidal crystal templating for reversible co_2_ capture. Adv. Funct. Mater. 23, 4720–4728 (2013).

[b27] Xing R. . Novel solid acid catalysts: sulfonic acid group-functionalized mesostructured polymers. Adv. Funct. Mater. 17, 2455–2461 (2007).

[b28] Meng Y. . Ordered mesoporous polymers and homologous carbon frameworks: amphiphilic surfactant templating and direct transformation. Angew. Chem. Int. Ed. 44, 7053–7059 (2005). 10.1002/anie.200501561 16222652

[b29] Zhang F. . A facile aqueous route to synthesize highly ordered mesoporous polymers and carbon frameworks with Ia*3*^−^d bicontinuous cubic structure. J. Am. Chem. Soc. 127, 13508–13509 (2005). 16190709 10.1021/ja0545721

[b30] Zhang X. . Robust hybrid raspberry-like hollow particles with complex structures: a facile method of swelling polymerization towards composite spheres. Soft Matter. 10, 873–881 (2014). 24837153 10.1039/c3sm52739e

