# Supplementary material for: Hierarchical Porous Interlocked Polymeric Microcapsules: Sulfonic Acid Functionalization as Acid Catalysts
Source: Sci Rep. 2017 Mar 16;7:44178. doi: 10.1038/srep44178 (PMC5353599; doi:10.1038/srep44178)
Supplement: Supplementary Information [file srep44178-s1.doc]

**Supporting Information**

Hierarchical Porous Interlocked Polymeric Microcapsules: Sulfonic Acid Functionalization as Acid Catalysts

Xiaomei Wang, Jinyan Gu, Lei Tian, Xu Zhang *

Department of Polymer Science and Engineering

Hebei University of Technology, Tianjin 300130, P.R. China

E-mail: xuzhang@hebut.edu.cn

**Figure S1**. The PS distribution plots of molecular weight.


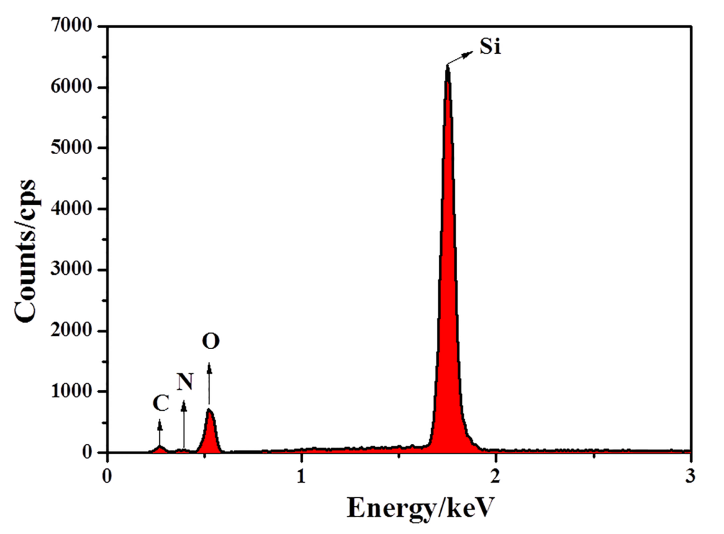


**Figure S2.** The EDX spectrum of BITS modified SiO_2_ opal.


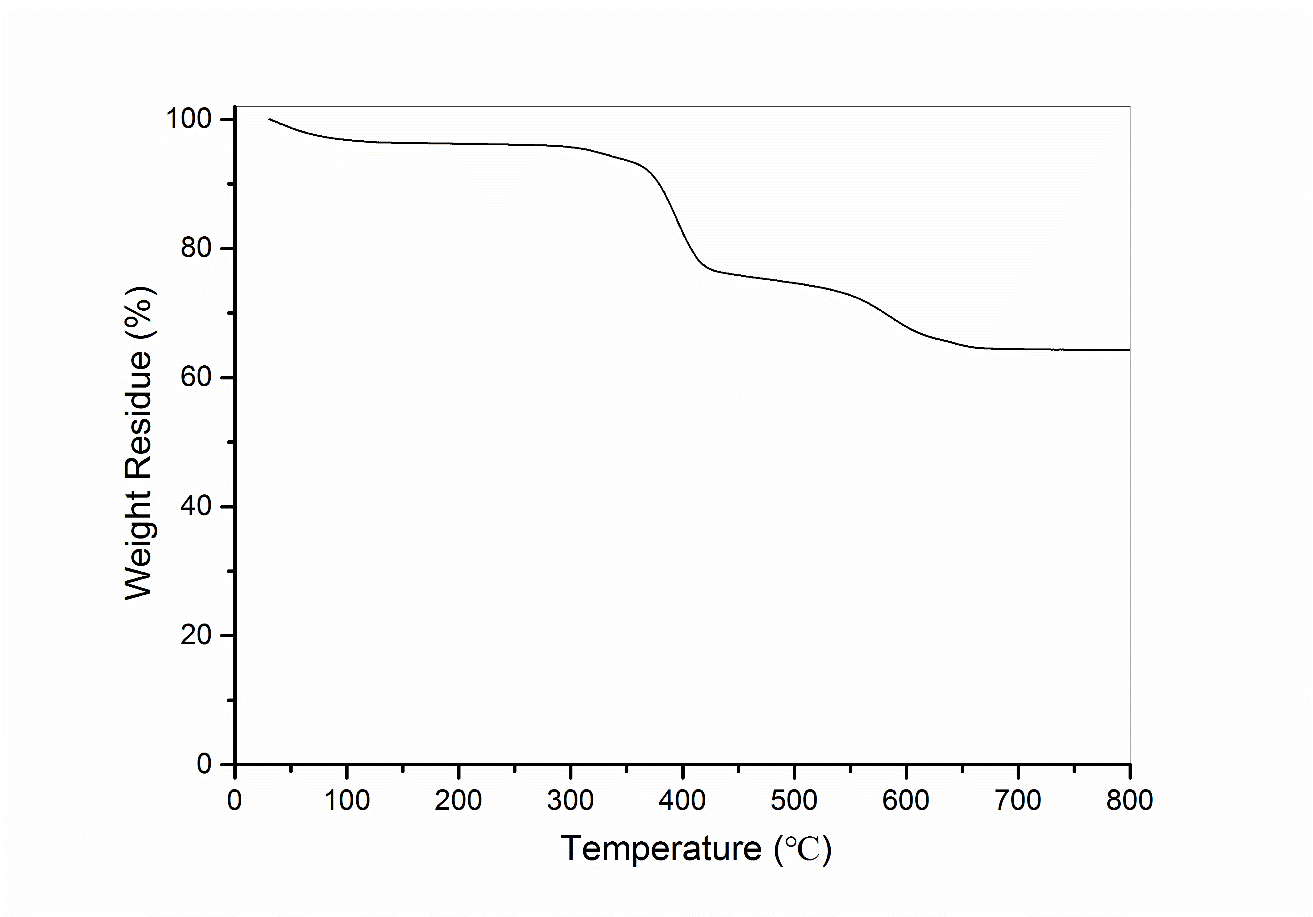


**Figure S3**. The TGA curve of the SiO_2_@LPS composite opal.


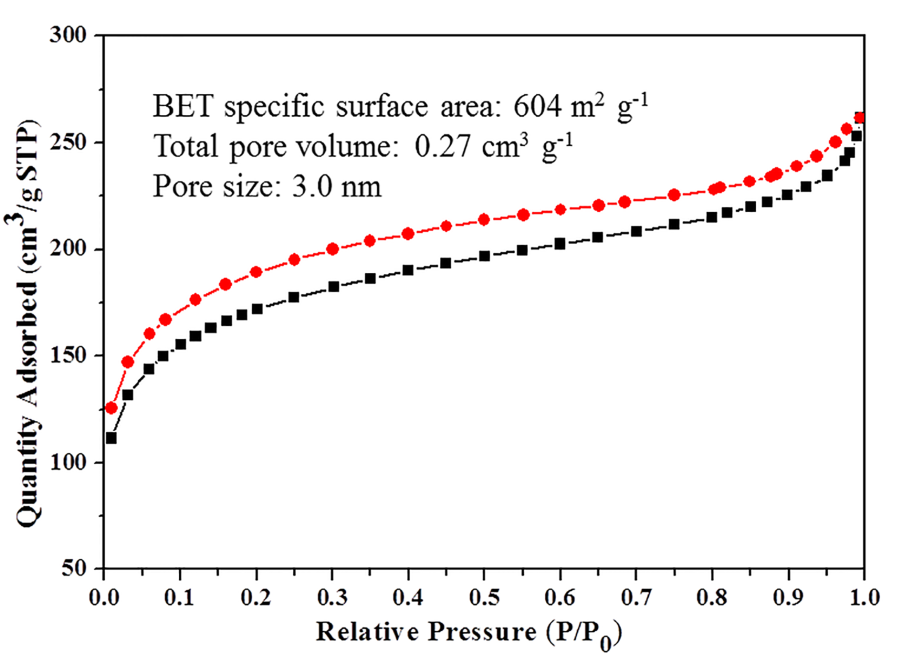


**Figure S4.** The nitrogen adsorption-desorption isotherms of HPIM-HCL-PS. Surface area was calculated using the BET equation. Peak pore diameter and pore volume were calculated using the BJH model.


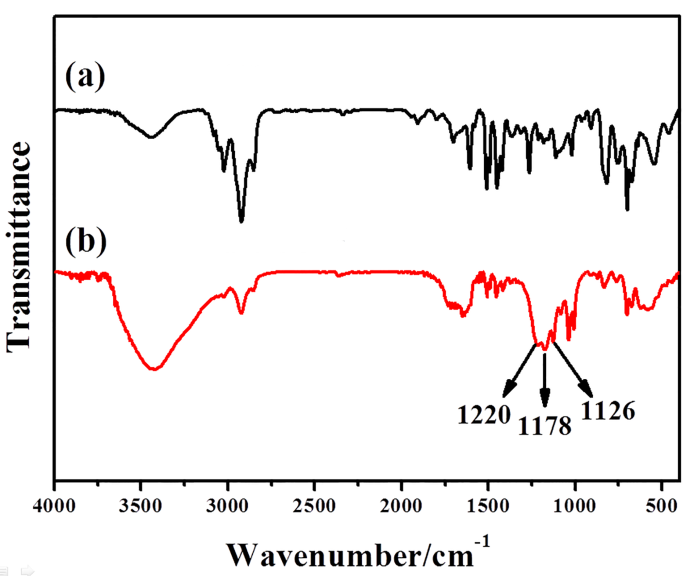


**Figure S5.** FT-IR spectra of (a) HPIM-HCL-PS and (b) HPIM-HCL-SPS.


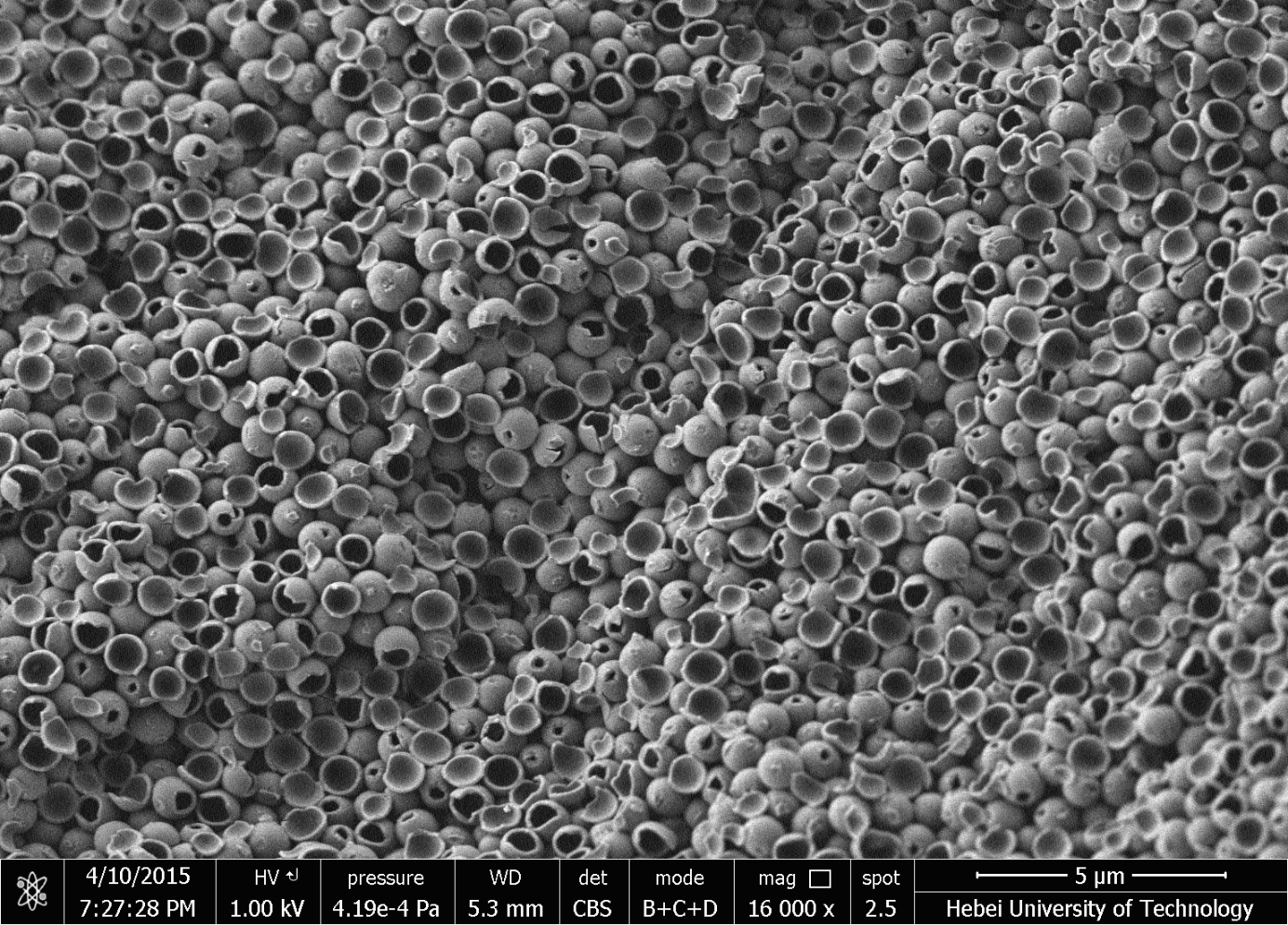


**Figure S6.** SEM image of the HPIM-HCL-SPS after five catalytic cycles.


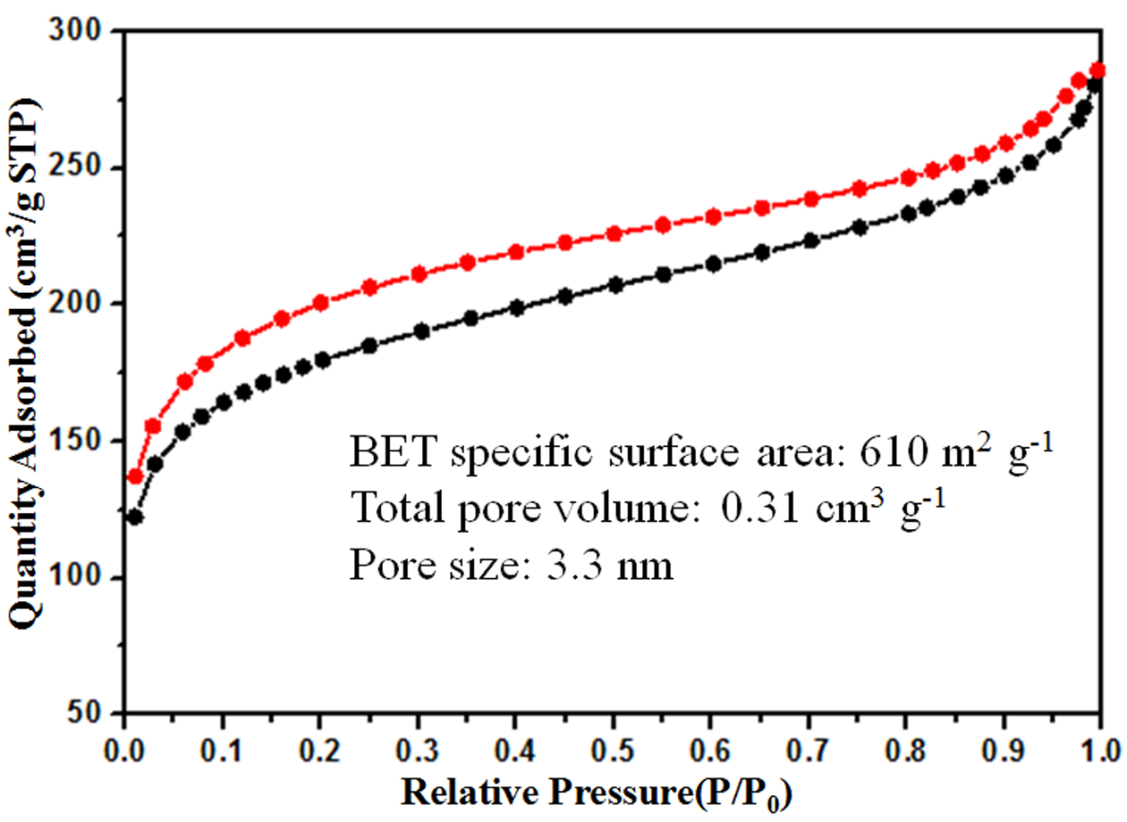


**Figure S7.** The nitrogen adsorption-desorption isotherms of HPIM-HCL-PS after five catalytic cycles. Surface area was calculated using the BET equation. Peak pore diameter and pore volume were calculated using the BJH model.
